# Unexpected exacerbation of cryptococcal meningitis after unilateral adrenalectomy in a PMAH patient: a case report and literature review

**DOI:** 10.1186/s12902-023-01457-5

**Published:** 2023-09-18

**Authors:** Lu Tan, Lianling Zhao, Guangmin Tang, Yan Ren, Haoming Tian, Tao Chen

**Affiliations:** 1 Department of Endocrinology and Metabolism, Adrenal Center, West China Hospital of Sichuan University, 37 GuoXue Lane, Chengdu, Sichuan 610041 P. R. China; 2 Center of Infectious Diseases, West China Hospital, Sichuan University, Chengdu, Sichuan 610041 P. R. China

**Keywords:** Primary bilateral macronodular adrenal hyperplasia, Cryptococcal meningitis, Cushing’s syndrome, Immune reconstitution inflammatory syndrome, Case report

## Abstract

**Background:**

Primary bilateral macronodular adrenal hyperplasia (PMAH) combined with infection by an opportunistic pathogen is complicated. Clinical evidence on managing PMAH patients with infections by opportunistic pathogens is insufficient.

**Case presentation:**

A 66-year-old male was admitted with bilateral adrenal masses and was diagnosed with PMAH. Fever and disturbance of consciousness appeared after laparoscopic left adrenalectomy. Cryptococcal meningitis was confirmed by cerebrospinal fluid (CSF) culture. The exacerbation of his medical condition was suspected to result from immune reconstitution inflammatory syndrome (IRIS), and he had been treated with antifungal therapy and glucocorticoid replacement, but he responded poorly and eventually died of multiorgan failure. We summarized the clinical observations of 12 Cushing's syndrome (CS) patients infected by Cryptococcus. Seven out of nine patients who were treated for cryptococcus infection before receiving CS survived, while three patients treated for cryptococcus infection after CS treatment developed signs of IRIS and eventually died.

**Conclusion:**

Cushing's syndrome, complicated with cryptococcal infection, has a high mortality rate, mainly when IRIS emerges. Carefully identifying the presence of the suspected infection, and controlling cryptococcal infection before removing the culprit adrenals could be the rational choice.

## Background

Primary bilateral macronodular adrenal hyperplasia (PMAH) is a rare cause of Cushing's syndrome (CS), and it is estimated to represent less than 2% of cases [1]. PMAH presents with bilateral adrenal nodules accompanied by hypercortisolemia and corresponding clinical manifestations. It progresses very slowly, and usually no typical cushingoid manifestations develop during the initial stage of the disease. PMAH patients exhibit severe immunosuppression and high sensitivity to infection by opportunistic pathogens. When immune reestablishment occurs after cortisol ablation treatment, such as adrenalectomy, immune reconstitution inflammatory syndrome (IRIS) might occur and cause aggravation of infections by opportunistic pathogens. In this report, we present a patient with clinically and genetically confirmed PMAH whose condition unexpectedly worsened after unilateral adenectomy, which was caused by IRIS related to rapid cortisol ablation and occult cryptococcal meningitis. To understand this complicated condition better, we further summarized the clinical observations of 12 Cushing's syndrome (CS) patients infected by Cryptococcus.

## Case presentation

A 66-year-old male complaining of limping and dizziness was observed to exhibit bilateral adrenal nodules by an abdominal computed tomography (CT) scan. He exhibited hypertension, hypokalemia, symptomatic epilepsy, chronic hydrocephalus, and systemic rash for years. He took 5 mg amlodipine besylate to control blood pressure and 1.5 g levetiracetam (Keppra) for epilepsy daily. On physical examination, his blood pressure was 154/89 mmHg, with mild signs of Buffalo hump, moon face, thin skin, slender limbs, and central obesity.

**Fig. 1:**
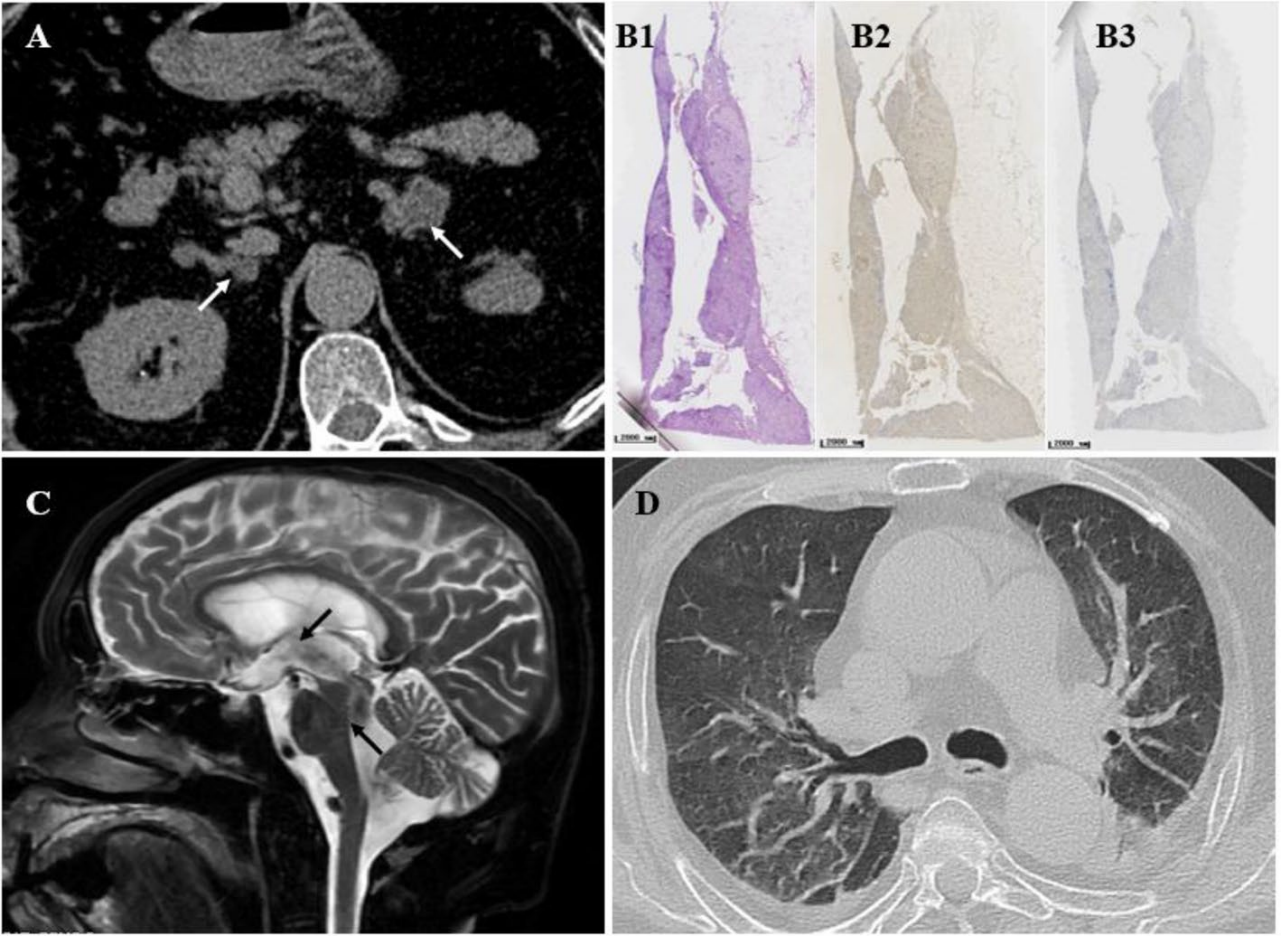
Imaging features of the patient's adrenal lesions and cryptococcal meningitis and immunohistochemistry of adrenal lesions. **A**: Adrenal CT showed multiple nodules and masses in the bilateral adrenal glands (white arrow). The largest tumor was on the left side, approximately 4.3 × 4.4 cm, with noticeable enhancement. **B**: Histology and immunohistochemistry (IHC) of adrenal tissues. Hematoxylin and eosin (HE) staining(B1) showed multiple nodular hyperplasia with adenomatoid nodules, immunohistochemistry of CYP11B1 was positive (B2) and CYP11B2 was negative (B3). Scale bar = 2000 μm. **C**: Head MRI showed bilateral lateral ventricles, the third and fourth ventricles were enlarged, and the meninges were thickened. **D**: Chest CT show Bilateral scattered inflammation in the lungs and no signs indicated Cryptococcal pneumonia

A laboratory examination revealed hypokalemia, a lack of rhythmicity in plasma cortisol levels, increased urine-free cortisol (UFC) levels after 24 h, a positive overnight 1 mg dexamethasone suppression test result, and low plasma ACTH levels (Table 1). Adrenal enhancement CT showed that the bilateral adrenal had several nodules, among which the largest (4.3 × 4.4 cm) was significantly enhanced and located on the left side (Fig. 1a). Dual energy X-ray absorptiometry results indicated osteoporosis.

**Table 1:** A laboratory and endocrinological data

Test		Result	Reference range
Cortisol	8:00	492.0	133–537 nmol/L
	24:00	483.0	< 50 nmol/L
	1 mg DST	462.0	< 50 nmol/L
	UFC (first)	1895.2(2.1L)	20.26–127.55 μg/24 h
	UFC (second)	2582.8(3.1 L)	
ACTH	8:00	< 1	5.0–78.0 (ng/L)
DHEA-S		0.620	0.91–6.76 (umol/L)
Cell of CSF	nuclear cells	190 × 10^6/L	0
	mononuclear cells (%)	97%	
	morpho nuclear leukocytes (%)	2.0%	
Biochemistry of CSF	Microalbumin(g/L)	2.69	0.15–0.45
	Glucose (mmol/L)	1.92	2.5–4.4
	Chlorine(mmol/L)	112	120–130
Fungal culture of CSF	Cryptococcus neoformans	Positive	Negative
Bacteria culture and tuberculosis culture of CSF		Negative	Negative
The autoimmune antigen of CSF	anti-NMDA antibody IgG	1:32	

*DHEA-S* Dehydroepiandrosterone-sulphate, *24 h-UFC* twenty-four hours urine-free cortisol, *CSF* Cerebrospinal fluid, *NMDA* N-methyl-D-aspartate receptor

He was initially diagnosed with PMAH. After a comprehensive assessment, he underwent laparoscopic left adrenalectomy without adrenal-vein sampling (AVS), since the left side lesion was larger (4.3 × 4.4 cm). Postoperative pathology was consistent with adrenocortical nodular hyperplasia with adenomatoid nodules, and immunohistochemistry assay showed the surgical sample was positive for CYP11B1(Merck Millipore). Gene sequencing of DNA from peripheral white blood cells suggested an ARMC5 gene mutation (chr16:31477527 c.2125(exon 6) C > T, Joy Orient Translational Medicine Research Center Co., Ltd).

On the first day after the surgery, the serum cortisol levels were significantly reduced to 123 nmol/L. Potassium supplementation and hydrocortisone replacement therapy were given. The hydrocortisone dose was gradually tapered from 300 mg per day to 50 mg per day. The patient's condition improved gradually. However, on the 14^th^ day after the left adrenalectomy, the patient developed low-grade fever (37.5℃), limb fatigue, and low mood and appetite. Assessments of serum electrolyte levels suggested hyponatremia (133.5 mmol/L) and hypokalemia (3.45 mmol/L), and the levels of inflammatory markers (C-reactive protein (CRP), procalcitonin, and IL-6) were slightly elevated. Chest CT indicated scattered inflammation in both lungs, and no signs indicated Cryptococcal pneumonia. A magnetic resonance imaging (MRI) examination of the head showed meningeal thickening and massive hydrocephalus, which affected the bilateral lateral ventricle, third ventricle, and fourth ventricle (Fig. 1b). The patient's condition gradually deteriorated, including urinary incontinence, difficulty moving, and even confusion. During lumbar puncture, an increased intracranial pressure (higher than 200 mmH_2_O) was observed, with pale-yellow, nonflocculent cerebrospinal fluid (CSF). After 20 mL of CSF was drained, the patient's consciousness slightly improved the next day. A laboratory examination confirmed the diagnosis of cryptococcal meningitis (Table 2). After consultation with a neurosurgeon and infectious diseases specialist, the patient was administered antifungal therapy containing amphotericin B (gradually increased from 5 to 30 mg) with 5-fluorocytosine 1.5 g q.i.d. and continuous lumbar drainage of fluid (LCDF) to drain the CSF adequately. Hydrocortisone supplementation and other supporting treatments were applied at the same time. His condition improved temporarily but deteriorated, and he died of multiple organ failure.

**Table 2:** Diagnosis, treatment, and outcomes of CS patients with cryptococcus infection in the current study

Study’s ID	Age	Sex	UFC (ug/d)	F (µg/dL)	CS Subtype and Treatment	cryptococcus infection sites and treatment	Treatment timing for Cryptococcus infection and CS	IRIS	Outcome
Kramer M [2]	62	F	NA	19.3	CD; pituitary surgery	pulmonary; AMPH-B + FU	anti-cryptococcus infection first	N	Relieved
Kramer M [2]	32	F	NA	29.8	CD; pituitary surgery	pulmonary; AMPH-B + FU	anti-cryptococcus infection first	N	Relieved
Yamagami, K [3]	47	M	NA	73	CD; pituitary surgery	pulmonary; Pulmonary lobectomy	anti-cryptococcus infection first	N	Relieved
Zhao, Y [4]	48	F	813.5	17.0	CD; pituitary surgery	pulmonary; FLC 400 mg/d *3 m	anti-cryptococcus infection first	N	Relieved
Tani, Y [5]	75	F	1706	31.6	EAS; pulmonary lobectomy	pulmonary; Pulmonary lobectomy	synchronous	N	Relieved
Zhang D, [6]	44	M	NA	19.7	EAS; pulmonary lobectomy	pulmonary; Pulmonary lobectomy	synchronous	N	Relieved
SBritton, S [7]	24	F	NA	NA	ACS; MET 1250 mg/d *8 m, Uni-ADX	**CM**; FU	synchronous	N	Relieved
Cheol-In K [8]	39	F	385.7	NA	CD; KCZ & OCT	**CM**, skin, and blood; AMPH-B	anti-cryptococcus infection first	N	**Died**
Lacativa, P G [9]	24	M	NA	56.3	CD; pituitary surgery	pulmonary; None	–-	**Y**	**Died**
Thangakunam, B [10]	52	M	NA	72	CD; KCZ	pulmonary; AMPH-B	anti-cryptococcus infection first	N	**Died**
Goto, T [11]	85	F	NA	74.7	EAS; MET	pulmonary; TMP-SMX & AmBisome	CS treatment first	**Y**	**Died**
The present study	66	M	2582.8	17.8	PMAH; Uni-ADX	**CM**; AMPH-B and FC	CS treatment first	**Y**	**Died**

*NA* Not available, *F* cortisol, *IRIS* Immune reconstitution inflammatory syndrome, *CD* Cushing’s disease, *EAS* Ectopic ACTH syndrome, *PMAH* Primary bilateral macronodular adrenal hyperplasia, *CM* cryptococcal meningitis, *MET* Metyrapone, *KCZ* ketoconazole, *OCT* octreotide, *Uni-ADX* unilateral adrenalectomy, *FU* fluorouracil, *FC* fluorocytosine, *AMPH-B* amphotericin B, *AmBisome* liposomal amphotericin B, *TMP-SMX* trimethoprim-sulfamethoxazole, *FLC* fluconazole, *PAs* pituitary adenomas

## Literature review

The search strategy used to comprehensively review infections by opportunistic pathogens in Cushing's syndrome patients involved identifying relevant and available articles for PMAH studies published before 2020 on MEDLINE (PubMed) and the Chinese Biomedical Database. Original research, case reports, case series, or published review articles were included. Medical subject headings (MeSH) terms included "ACTH independent macronodular adrenal hyperplasia," "Cushing's syndrome," "cryptococcal," and "Immune reconstitution inflammatory syndrome." Studies that analyzed cases of exogenous Cushing's syndrome without any treatment for Cushing's syndrome and patients with positivity for plasma human immunodeficiency virus (HIV) antibodies were all excluded.

Between 1975 and 2020, 18 patients were diagnosed with Cushing's syndrome and cryptococcal infection and included in the study. The median age was 45.4 years (range 20–85), and 55.5% were female. Serum cortisol results were provided for sixteen cases, with a mean of 42.47 µg/dL (range 17–74.7 µg/dl). The 24-h UFC average of the 10 cases was 1981.9 µg/d (range, 374–4500 µg/d). The most common cause of Cushing's syndrome was Cushing's disease (66.6%). The most common infection site was the lung (83.3%). There were only 3 cases of cryptococcal meningitis with no evidence of infections in other organ. Approximately 38.9% (7/18) of the patients were cured of hypercortisolemia and cryptococcal infection after treatment.

To better understand the factors associated with poor prognosis, we further analyzed 12 cases with detailed courses and characteristics, and six patients with insufficient data were omitted. Among the 12 patients, seven were diagnosed with CD, three with ectopic adrenocorticotropic hormone syndrome (EAS), and two with adrenal Cushing syndrome (ACS). Six of the seven CD patients exhibited complications with pulmonary cryptococcus infection and another with disseminated cryptococcal infection (CM, skin, and blood). Four of them were treated for cryptococcus infection before CS treatment and survived; one without any cryptococcus infection treatment developed IRIS and died, and the other two were treated for cryptococcus infection first, but one had disseminated cryptococcal infection, while the other had multiple organ failure and mixed bacterial infection. All three patients with EAS exhibited complications with pulmonary cryptococcus infection; two who were treated for cryptococcus infection and EAS simultaneously by surgery survived, while the other who was treated for EAS first developed signs of IRIS and died. The last two ACS patients (1 adenoma patient and 1 PMAH patient in the present report) exhibited complications with CM, and one treated for CM and CS simultaneously was cured, while the patient in the present study was treated for CS first and died.

All three patients (1 CD, 1 EAS, and 1 ACS) who developed IRIS-related symptoms died after a rapid reduction in hypercortisolism status. In addition to the patient we presented, one of the other two patients was a 24-year-old Brazilian male who underwent pituitary surgery for CD and received oral prednisone (10 mg per day) after discharge. Soon after discharge, he developed fatigue, dizziness, hypotension, and vomiting and finally died of respiratory failure, which was confirmed by autopsy as being caused by cryptococcus infection. The third patient was an 85-year-old Japanese woman treated with metyrapone for ectopic ACTH syndrome first and with amphotericin B and trimethoprim-sulfamethoxazole for cryptococcal infection two weeks later. She developed fever, fatigue, respiratory failure, and disturbance of consciousness and finally died of acute respiratory distress syndrome (ARDS) due to pulmonary cryptococcus infection (Table 2).

## Discussion

The patient described in the present report suffered from PMAH with a previously reported pathogenic ARMC5 gene mutation (c.2125C > T, exon 6). He developed overt Cushing syndrome due to prolonged clinical course. The prominent clinical manifestation involved hypertension and hypokalemia, complicated with epilepsy and unexplained chronic hydrocephalus. Unexpectedly, his condition worsened instead of improved after left adrenalectomy to reduce autonomous cortisol secretion. Subsequent work-up confirmed the diagnosis of cryptococcal meningitis, which caused hydrocephalus and mental status changes, which might have occurred several years before this admission. It was confusing that this opportunistic infection seemed to be exacerbated by the ablation of excessive cortisol. Moreover, even after treatment with the potent anti-fungus regimen and glucocorticoid supplement, the patient died four months later. he patient's death may have been attributed to the failure of the test for cryptococcal infection. In conjunction with the evidence from the existing literature, we propose that the patient's death could be plausibly ascribed to the failure to detect a central cryptococcus infection, primarily stemming from immunosuppression induced by CS. This oversight subsequently precipitated the onset of IRIS after the adenectomy.

Chronic hypercortisolism induces various alterations in the immune system and puts the body in an immunosuppressive state [12, 13], frequently leading to severe clinical complications such as sepsis and opportunistic infections [14]. Moreover, chronic hypercortisolism inhibits the activity of many different immunomodulatory factors (IL-1, IL-6, TNF α, etc.) and immune cells, which leads to a chronic low-grade inflammatory response and a selectively impaired immune response [14]. After relieving a high cortisol state, the previously suppressed immune system reactivates and even becomes overactivated, leading to autoimmune diseases development [15], uncovering preexisting sarcoidosis [16], promoting disease deterioration and increased mortality, a phenomenon referred to as IRIS [17, 18].

PMAH is a vastly heterogeneous condition, typified by the excessive secretion of cortisol from large nodules in both adrenal glands. For patients exhibiting overt CS, the treatment typically preferred is bilateral adrenalectomy, followed by lifelong replacement of adrenocortical hormones. However, to mitigate the necessity for lifelong hormone replacement, unilateral adrenalectomy has recently emerged as an advocated alternative. As elucidated in a study by Vassiliadi DA et al., an initial remission was documented in more than 90% of PMAH cases following unilateral adrenalectomy, with approximately one-third of these patients subsequently manifesting symptoms of adrenal insufficiency [19]. Such findings signal the existence of asymmetrical cortisol production within the bilateral adrenal lesions in PMAH patients.

In the present case, a significant reduction in serum cortisol levels from 492 to 123 nmol/L was observed post-unilateral adrenalectomy, accompanied by the development of symptoms indicative of adrenal insufficiency such as fever and decreased appetite. This patient scenario suggests that the removed left adrenal lesion had a greater cortisol production rate compared to the right adrenal lesion. Following the surgical removal of the left adrenal gland, the subsequent adrenal insufficiency instigated the IRIS, exacerbating the patient's condition and ultimately contributing to the patient's death.

Currently, there are no sufficient observational data to generate a treatment strategy for IRIS after cortisol ablation treatment for CS. To this end, we compared the therapeutic measures and outcomes of 12 patients with Cryptococcus infection complicated with CS through a systematic review of published literature. The systematic analysis of these 12 patients showed that hypercortisolemia could complicate cryptococcal infection for a long time. Nevertheless, only 50% of patients would be screened for cryptococcal infection when CS was diagnosed, which was consistent with the notion that hypercortisolemia masked the inflammatory response by cryptococcal infection [14].

All surviving patients, even two with EAS, were treated for cryptococcal infection first or simultaneously. Three of the five patients who died who were treated for CS first died. The other two patients who died were first treated for cryptococcal infection, but both had a severe cryptococcal infection, developed multiple organ failure, and had no chance for effective treatment for CS. These results indicated that treatment targeting cryptococcal infection first or simultaneously with CS was the critical point for a good prognosis.

All three patients who died who had been treated for hypercortisolism first had not been suspected of having cryptococcal infection at the time of CS diagnosis and had been recommended for surgical treatment for CS. All of the patients developed a high fever, limb weakness, and the rapid progression of infection within a short period (2–14 days) after hypercortisolemia ablation treatment and eventually died. Such deterioration was considered IRIS-related and was speculated to be related to the steep fall in cortisol levels. Regrettably, none of the patients was tested for markers of IRIS, such as IFN-γ, CD4 + T cells, and type I interferons (IFN I) [20]. However, there is other evidence consistent with our findings. For instance, Karlijn van Halem et al. analyzed eighteen CS patients with pneumocystis pneumonia and found that all the conditions of the patients, such as fever, fatigue, poor appetite, respiratory failure, and consciousness disturbance, were exacerbated several days after cortisol ablation treatment, and only 6 survived [21]. Other evidence indicated that prophylaxis for pathogens and gradual lowering rather than rapid reduction of cortisol levels might be beneficial for survival in CS patients with different opportunistic infections [22–25]. This evidence suggests that IRIS might occur after rapid cortisol reduction without predefined targeted treatment for opportunistic infection, and IRIS might be a considerable risk factor for poor prognosis in CS patients with opportunistic infection.

The limitation of the present study was that we did not evaluate the patient’s exact immune status before and after cortisol-ablation surgery. The symptoms that were exacerbated after adrenalectomy were presumed to be related to IRIS. Further studies are needed to address this issue.

## Conclusion

PMAH with cryptococcal meningitis is rare but occurs. Early treatment for Cryptococcus infection might improve the consequences of IRIS. More attention should be given to screening for cryptococcal infection in CS patients.

## Data Availability

The datasets used and/or analysed during the current study are available from the corresponding author on reasonable request.

## Abbreviations

IRIS: Immune reconstitution inflammatory syndrome
CD: Cushing’s disease
EAS: Ectopic ACTH syndrome
PMAH: Primary bilateral macronodular adrenal hyperplasia
CM: Cryptococcal meningitis
